# Cecal volvolus herniation through the Winslow Foramen: a case report and literature review of surgical management

**DOI:** 10.1093/jscr/rjae325

**Published:** 2024-05-26

**Authors:** Marco Gramellini, Folco Solimene, Moritz Menz, Lorenz Hinterleitner, Stefan Breitenstein, Patryk Kambakamba

**Affiliations:** Department of Surgery, Kantonsspital Winterthur, Brauerstrasse 15, Postfach, Winterthur 8401, Switzerland; Department of Surgery, Kantonsspital Winterthur, Brauerstrasse 15, Postfach, Winterthur 8401, Switzerland; Department of Surgery, Kantonsspital Winterthur, Brauerstrasse 15, Postfach, Winterthur 8401, Switzerland; Medical University of Vienna, MedUni Vienna, Spitalgasse 23, Vienna 1090, Austria; Department of Surgery, Kantonsspital Winterthur, Brauerstrasse 15, Postfach, Winterthur 8401, Switzerland; Department of Surgery, Kantonsspital Winterthur, Brauerstrasse 15, Postfach, Winterthur 8401, Switzerland

**Keywords:** cecal volvolus, Winslow hernia, Winslow Foramen, rare disease

## Abstract

Both cecal volvolus and Winslow hernia are rare clinical presentations accounting for 1–1.5 and 0.08% of bowel obstructions. The combination of the two phenomena has been described so far in 13 case reports. Our patient underwent laparotomy with lesser Sac opening, manual hernia reduction, right hemicolectomy and partial Foramen closure with two simple stitches of PDS 4.0. Due to the scarcity of literature guidelines are not available, the intraoperative state of the tissues and the likelihood of a hernia recurrence play a decisive role in surgical management.

## Introduction

A cecal volvolus (CV) is a twisting or axial rotation of the cecum and accounts for 1–1.5% of all intestinal obstructions [1]. Even rarer are internal hernias, also accounting for only 1% of Bowel Obstructions. The Foramen of Winslow Hernia (FWH) accounts for 8% of internal Hernias and the cecum is the herniated organ only in one-third of the cases [2]. Both clinical presentations are associated with a mortality rates up to 30 and 49%, respectively if not promptly recognized and treated [3, 4]. The combination of CV and FWH is an extremely rare Phenomenon and has to our knowledge been reported only 13 times in the literature. An excessive viscera mobility with persisting ascending mesocolon, a Winslow Foramen (WF) of more than 3 cm and changes in intra-abdominal pressure are the advocated pathological mechanisms [4]. Data regarding its management are scarce and no guidelines are available. In this case report, we describe our procedure and we compare it with the surgical management in the other reported cases.

## Case report

**Figure 1:**
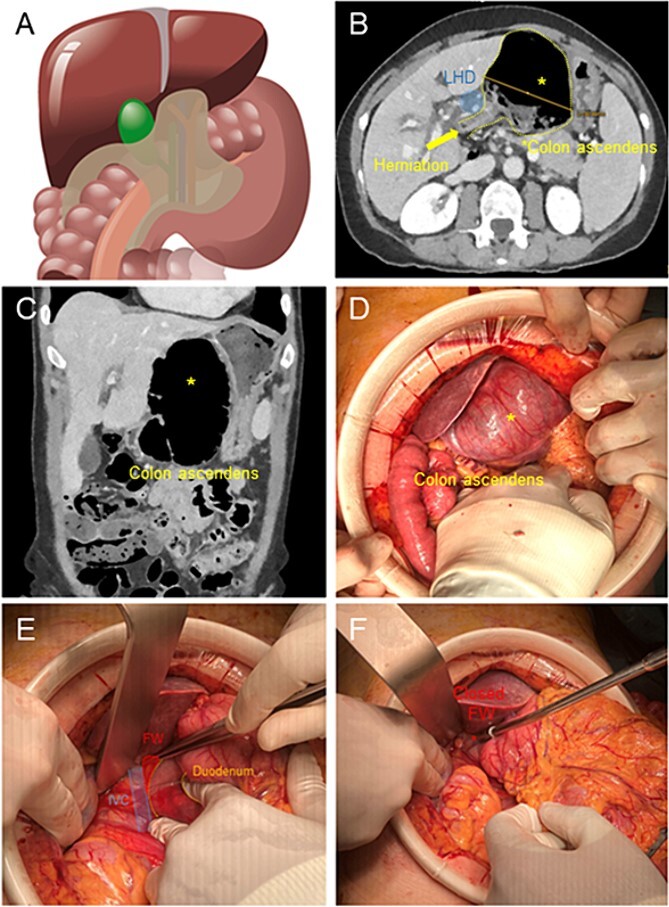
(A) Illustration of cecal herniation through WF as shown in CT scan. (B) Dilated colon ascendens behind the Ligamentum Hepatoduodenale (LHD) in the CT Scan. (C) The coronary view shows the dilated colon. (D) Intraoperative hernia presentation after laparotomy, the dilated colon presents a serosal tear (*) in its surface. (E) After reduction of hernia, exposure of WF, with inferior vena cava below and Duodenum to the right. (F) Closure of WF with interrupted suture with p-Dioxanon (PDS 4-0).

A 68-year-old woman presented in the emergency department of our hospital with epigastric pain irradiated to the back without vomiting or fever. Past clinical history included Hashimoto Thyroiditis and Polycitaemia Vera, with no previous surgical operations. The clinical examination showed a soft abdomen but with bloating, defence reaction and abdominal pain in epigastrium. Blood testing revealed 9.96 G/L leucocytes and 1 mg/l C-reactive protein. We performed a computer tomography (CT) of the abdomen, which identified a retrogastric volvulated cecum (Fig. 1B and C) and the indication to surgery was given.

We performed a 12-cm laparotomy, which allowed us to confirm the diagnosis (Fig. 1D). We incised the thin lesser sac, performed a manual derotation of the cecum and cautiously successfully pulled laterally the herniated viscera through the WF (Fig. 1E). The cecum appeared massively dilated with serosal tears. The whole ascending colon appeared lax and hypermobile. Therefore we performed a right hemicolectomy with a stapled side-to-side ileo-colic anastomosis and mesenterial closure. To prevent the recurrence of visceral herniation through the WF, we applied two simple stitches in PDS 4.0 in the inferior portion of the WF aiming to restore its normal diameter (Fig. 1F).

Further postoperative course was uneventful and the patient was discharged on the fifth POD. At 1-year follow-up, the patient hasn’t shown any evidence of hernia recurrence and is feeling well.

## Discussion

The combination of FWH and CV is an extremely rare clinical presentation. 12 case reports in English language are reported on Pubmed. Their features as well as that of our case are summarized in Table 1. Out of 13, 12 are female patients, age range: 55–82 years. These findings are coherent with literature as CV is more common in females in this age range [5], although FWH appears to be predominant in males [4]. Typical presentations include abdominal pain with or without vomiting and bloating in the upper abdominal quadrants. CT was the preferred imaging, able to identify the bloated ectopic cecum in the lesser sac and the possible twist of the ileocolic vessels. In the case of Lawson *et al.* [6] ischemic changes in the liver due to the complete occlusion of the portal vein were also reported, highlighting how a bloated bowel loop herniated through FWH can as well impact the hepato-duodenal Ligament’s structures.

**Table 1:** Clinical presentation and treatment of CV through the FWH

	**Clinical presentation features**				**Surgical management**				
	**Male/female**	**Patient age (y)**	**Symptoms**	**Lab tests**	**Laparotomy**	**Lesser sac incision**	**Hernia reduction**	**Bowel resection**	**Foramen closure**
Morioka *et al.* 1970 [14]	F	67	Abdominal pain		yes	no	yes	no	no
Ray et al 2009 [15]	F	55	Abdominal pain Vomiting	unremarkable	yes	no	yes, manual	yes	yes with omental flap
Makarawo et al 2014 [12]	F	75	Abdominal pain Vomiting	unremarkable	yes	yes	yes, manual	no, cecopexy	yes with omental flap
Kamyab *et al.* 2016 [10]	F	67	Upper abdominal pain Nausea Vomiting		yes	no	yes, through Kocher Maneuver	yes	no
Nguyen *et al.* 2017 [7]	F	73	Abdominal pain Nausea Vomiting	slightly elevated WBC	yes	yes	yes, through cecal enterotomy	yes	no
Patel *et al.* 2017 [11]	M	62	Upper abdominal pain	WBC 15 G/l CRP 300 mg/l	yes			yes + diverting Ileostomy	
Lawson *et al.* 2017 [6]	F	56	Upper abdominal Pain	unremarkable	yes	no	yes, manual	yes	no
Cho *et al.* 2019 [8]	F	56	Abdominal pain Nausea Vomiting	WBC 35,8 x10^9^; CRP 360 mg/l	yes	yes	yes, through cecal enterotomy and lysis of adhesions	yes	no
Williams *et al.* 2021 [13]	F	63	Upper abdominal pain	WBC 12,9 K/uL;	yes	no	yes, manual	no, cecopexy	yes with omental flap
Chandhrasekar *et al.* 2022 [9]	F	82	Abdominal pain Nausea Vomiting	unremarkable	yes	yes	yes, through Kocher Maneuver and cecal enterotomy	yes	yes with omental flap
Carpenter *et al.* 2022 [2]	F	70	Abdominal pain Nausea Vomiting	WBC 16.6/mm^3^	yes	yes	no, the resection was performed before reduction	yes	yes, complete closure with Vicryl stitches + omental flap
Perabo *et al.* 2022 [1]	F	56	Lower abdominal pain Nausea		yes	no	yes, manual	yes	no
Our case	F	68	Upper abdominal pain	WBC 9,96 G/L; CRP 1 mg/ml	yes	yes	yes, manual	yes	yes, partial with PDS 4.0 stitches

In all cases, a straightforward laparotomy was performed due to the potential ischemic risk of the overly distended bowel suffering from both volvulation and incarceration. In 2019, Moris *et al.* [4] reported a series of 15 FWH of different organs (ileum, colon, gallbladder) successfully achieving a laparoscopic hernia reduction and, among them, only two cases required a bowel resection. However, we deem the applicability of laparoscopy in case of a volvulated organ through the WF limited, due to a more evident bowel distension hindering repositioning. Furthermore, a failed reposition implies a subsequent laparotomy with possible advanced necrotic state of the bowel due to the time delay.

The reduction of the hernia was achieved in 11 cases out of 12 (in 1 case not mentioned) and, in 6 cases, required the incision of the hepato-gastric ligament to perform a manual derotation of the bowel (such as in our case) or a cecal enterotomy to release the bowel content and reduce the swelling [7–9]. Kocher Manouver was also performed in two cases [9, 10]. In the case of Carpenter *et al.* [2], the bowel could not be reduced and right hemicolectomy was directly performed.

A right hemicolectomy was performed in 10 cases out of 13 to remove the ischemic pre-perforated cecum. Patel *et al.* [11] report the only case with a perforated cecum at diagnosis and in this case a diverting ileostomy was performed and at a later date reversed. Makarawo *et al.* and Williams *et al.* [12, 13] report two cases of cecal bascule, where the Cecum folds on itself rather than twisting on its vascular axis and resulting in ischemia. The resection could be spared and a cecopexy was performed to reduce the gut mobility and prevent the recurrence of CV.

Foramen closure as a prevention maneuver was performed in 6 cases out of 12 (in one case it is not mentioned) and was except in our case always performed with an omental flap. We preferred a partial closure with simple stitches to restore the standard anatomy. In the review of D. Moris [4] among the 15 operated patients, due to FWH, only 5 underwent a foramen closure but none of them experienced an FWH recurrence in a median follow-up of 3 months. No evidence so far demonstrates foramen closure results in decreased FWH recurrence, nor a preferable closure method (omental flap vs stitches).

Further evidence is required to establish guidelines for surgical management of this uncommon presentation. The status of the cecum and the likelihood of recurrence should guide the surgeon in the intraoperative decision making.
